# The Distance Between Mars and Venus: Measuring Global Sex Differences in Personality

**DOI:** 10.1371/journal.pone.0029265

**Published:** 2012-01-04

**Authors:** Marco Del Giudice, Tom Booth, Paul Irwing

**Affiliations:** 1 Department of Psychology, University of Turin, Torino, Italy; 2 Manchester Business School, University of Manchester, Manchester, United Kingdom; University of Bologna, Italy

## Abstract

**Background:**

Sex differences in personality are believed to be comparatively small. However, research in this area has suffered from significant methodological limitations. We advance a set of guidelines for overcoming those limitations: (a) measure personality with a higher resolution than that afforded by the Big Five; (b) estimate sex differences on latent factors; and (c) assess global sex differences with multivariate effect sizes. We then apply these guidelines to a large, representative adult sample, and obtain what is presently the best estimate of global sex differences in personality.

**Methodology/Principal Findings:**

Personality measures were obtained from a large US sample (N = 10,261) with the 16PF Questionnaire. Multigroup latent variable modeling was used to estimate sex differences on individual personality dimensions, which were then aggregated to yield a multivariate effect size (Mahalanobis *D*). We found a global effect size *D* = 2.71, corresponding to an overlap of only 10% between the male and female distributions. Even excluding the factor showing the largest univariate ES, the global effect size was *D* = 1.71 (24% overlap). These are extremely large differences by psychological standards.

**Significance:**

The idea that there are only minor differences between the personality profiles of males and females should be rejected as based on inadequate methodology.

## Introduction

The psychology of human males and females is marked by a complex pattern of similarities and differences in cognition, motivation, and behavior. Describing and quantifying sex differences is a crucial task of psychological science, and a source of lively controversy among researchers [1]–[12]. In this paper, we will consider the nature and magnitude of sex differences in *personality*. It is difficult to overstate the theoretical and practical importance of sex differences in personality; finding large overall differences would tell us that the sexes differ broadly in their emotional and behavioral patterns, rather than just in a few (and comparatively narrow) motivational domains such as aggression and sexuality.

### The Gender Similarities Hypothesis

The idea that the sexes are quite similar in personality – as well as most other psychological attributes – has been expressed most forcefully in Hyde's “gender similarities hypothesis” [9]. The gender similarities hypothesis holds that “males and females are similar on most, but not all, psychological variables. That is, men and women, as well as boys and girls, are more alike than they are different.” Hyde's paper has been remarkably influential; between 2005 and 2010, it has accumulated 247 citations in the Web of Knowledge database and 498 citations in Google Scholar (retrieved May 19^th^, 2011).

While the gender similarities hypothesis does not make specific predictions about personality, sex differences in personality were found to be “small” in Hyde's meta-analytic review. Specifically, Hyde found consistently “large” (*d* between .66 and .99) or “very large” (*d*≥1.00) sex differences in only some motor behaviors and some aspects of sexuality; “moderate” differences (*d* between .35 and .65) in aggression; and “small” differences (*d* between .11 and .35), or even differences close to zero (*d*≤.10) in the other domains she considered. Cohen's *d* is a standardized difference, obtained by dividing the difference between group means by the pooled within-group standard deviation. Assuming normality, a standardized difference *d*≤.35 implies that the male and female distributions overlap by at least 75% of their joint area. Even if conventional criteria for labeling effect sizes as “small”, “medium” and “large” have many limitations and should be used with great caution [2], [13], [14], this amount of overlap does indicate that the statistical distributions of males and females are not strongly differentiated. In fact, the nonoverlapping portion of the joint distribution becomes larger than the overlapping portion only when *d*>.85. For comparison, the criterion used by Hyde to identify “very large” sex differences (*d*≥1.00) corresponds to an overlap of 45% or less between the male and female distributions.

### The Evolutionary Psychology of Sex Differences

At the other end of the theoretical spectrum, evolutionary psychologists have emphasized how divergent selection pressures on males and females are expected to produce consistent – and often substantial – psychological differences between the sexes [1], [7], [15], [16]. By the logic of sexual selection theory and parental investment theory [17], [18], large sex differences are most likely to be found in traits and behaviors that ultimately relate to mating and parenting. More generally, sex differences are expected in those domains in which males and females have consistently faced different adaptive problems. For example, typical effect sizes in research on mate preferences range from *d* = .80 to 1.50, a finding consistent with this expectation [1], [19]. In contrast, similarities between males and females can be expected when the sexes have been subjected to similar selection pressures. Thus, the evolutionary approach to sex differences is consistent with a weak version of the gender similarities hypothesis [19], although the latter is stated so vaguely that it is extremely difficult to test empirically (e.g., how many “psychological variables” should be considered, and what is the appropriate index of similarity?).

Most personality traits have substantial effects on mating- and parenting-related behaviors such as sexual promiscuity, relationship stability, and divorce. Promiscuity and the desire for multiple sexual partners are predicted by extraversion, openness to experience, neuroticism (especially in women), positive schizotypy, and the “dark triad” traits (i.e., narcissism, psychopathy, and Machiavellianism). Negative predictors of promiscuity and short-term mating include agreeableness, conscientiousness, honesty-humility in the HEXACO model, and autistic-like traits [20]–[31]. Relationship instability is associated with extraversion, low agreeableness, and low conscientiousness [26], [29]–[31]. Finally, neuroticism, low conscientiousness, and (to a smaller extent) low agreeableness all contribute to increase the likelihood of divorce [32], [33].

In addition to their direct influences on mating processes, personality traits correlate with many other sexually selected behaviors, such as status-seeking and risk-taking (see e.g., [20], [34], [35]). Thus, in an evolutionary perspective, personality traits are definitely *not* neutral with respect to sexual selection. Instead, there are grounds to expect robust and wide-ranging sex differences in this area, resulting in strongly sexually differentiated patterns of emotion, thought, and behavior – as if there were “two human natures”, as effectively put by Davies and Shackelford [15].

### Aim of the Paper

Given the contrast between the predictions derived from evolutionary theory and those based on the gender similarities hypothesis, there is a pressing need for accurate empirical estimates of sex differences in personality. Of course, the existence of large sex differences would not, by itself, constitute proof that sexual selection had a direct role in shaping human personality. For example, Eagly and Wood [4] advanced an alternative theory in which selection is assumed to be responsible for physical, but not psychological differences between the sexes – the latter resulting from sex role socialization. Nevertheless, the accurate quantification of between-sex differences represents a necessary initial step toward an informed theoretical debate [36], and may eventually help researchers discriminate between alternative models of biological and cultural evolution [2].

The task of quantifying sex differences in personality faces a number of important methodological challenges. Indeed, all the studies performed so far suffer, to various degrees, from limitations that ultimately lead to systematic underestimation of effect sizes. In this paper, we discuss those challenges and present a set of guidelines for the accurate measurement of sex differences. We then apply our guidelines to the analysis of a large, representative US sample to obtain a “gold standard” estimate of global sex differences in personality, which turns out to be extremely large by any reasonable criterion.

### Methodological Challenges and Guidelines

In this section we review three key methodological challenges in the quantification of sex differences in personality. In doing so, we review the main empirical studies in this area and present the relevant effect sizes. However, the reader should keep in mind that our aim is to illustrate key methodological issues, not to present a comprehensive review of empirical research. In presenting univariate effect sizes (*d*), a positive sign indicates a male advantage, and a negative sign a female advantage.

#### Broad Versus Narrow Personality Traits

Personality traits can be organized in a hierarchical structure, from the broad and inclusive (e.g., extraversion) to the narrow and specific (e.g., gregariousness or excitement seeking). Researchers often focus on the Big Five, i.e., the broad “domains” of the five-factor model of personality (FFM [37]), which is at the same hierarchical level as the six factors of the HEXACO model [38], the five factors of the “alternative FFM” by Zuckerman and colleagues [39], and others. Up in the hierarchy, correlations between broad traits give rise to two “metatraits”, often labeled *stability* and *plasticity* [40], [41]. It may be even possible to identify a single, general factor of personality (the GFP or “Big One”) at the top of the hierarchy [42]–[45]. Right below the level of the Big Five, about 10–20 narrower traits can be identified; the ten “aspects” described by DeYoung and colleagues [46] and the fifteen primary factors in Cattell's 16PF [47], [48] fall in this category. At the lowest level are dozens of specific personality “facets”; questionnaires based on the FFM typically identify 30 to 45 such facets [46].

Choosing the proper level of description is a crucial challenge in the study of sex differences. Specifically, differences that are apparent (and possibly substantial) at a given descriptive level may become muted, or even disappear, when traits are aggregated into broader constructs at a higher hierarchical level. The effect is especially dramatic when sex differences in two narrow traits go in the opposite direction, canceling out one another at the level of a broader trait. For example, FFM extraversion has loadings on two narrower dimensions, warmth/affiliation (consistently higher in females) and dominance/venturesomeness (consistently higher in males). These two effects of opposite sign result in a small overall sex difference in extraversion, with females typically scoring (slightly) higher than males [49]–[54]. A similar pattern of crossover sex differences has been found in openness to experience, with males scoring higher on the “ideas” dimension and females on the “aesthetics” dimension of this trait [49], [54], [55]. Sex differences in Conscientiousness are also confined to just some of its components [50], [52].

Taken together, these findings make it apparent that measuring personality at the level of the Big Five hides some important differences between the sexes. Thus, in order to get the most accurate picture of sex differences, researchers need to measure personality with a higher resolution than that afforded by the Big Five (or other traits at the same hierarchical level). A corollary is that, when investigating sex and personality as predictors of a given outcome (such as health, self-esteem, and so forth), cleaner and more meaningful results are likely to obtain if personality is measured at the level that yields the most clearly sex-differentiated profiles. As traits become narrower, however, it also becomes more difficult to measure them with sufficient reliability, and the signal-to-noise ratio decreases. We provisionally suggest that the best compromise may be reached by describing personality with about 10–20 traits, i.e., at the hierarchical level immediately below that of the Big Five.

#### Observed Scores Versus Latent Variables

Theories of personality conceptualize traits as unobserved latent variables; as such, they are only imperfectly represented by scores on personality inventories. Typically, observed scores are contaminated by substantial amounts of specific variance and measurement error, leading to attenuated estimates of sex differences when such scores are used to compute effect sizes. Unfortunately, most studies of sex differences in personality have relied on observed scores, thus making the implicit (and incorrect) assumption that observed scores are equivalent to latent variables.

The contrast between latent, error-free effect sizes and observed-score effect sizes is strikingly illustrated by a recent study by Booth and Irwing [54]. On the 15 primary factors of the 16PF, observed-score effect sizes ranged from *d* = −1.34 to +.32, with an average absolute effect size 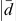 = .26 (within the bounds of “small” effects according to Hyde [9], and corresponding to a male-female overlap of 81%). When group differences on latent variables were estimated by multi-group covariance and mean structure analysis (MG-CMSA), effect sizes ranged from *d* = −2.29 to +.54, with an average absolute effect size 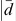 = .44 (a “moderate” effect by Hyde's criteria, corresponding to an overlap of 70%).

Estimating group differences on latent variables is clearly preferable to relying on observed scores, but this methodology depends on the assumption of *measurement invariance*, i.e., the assumption that the construct being measured is actually the same in both groups [56]–[58]. Booth and Irwing [54] found that between-sex invariance was violated for the five global scales of the 16PF (analogous to the Big Five), but satisfied for the 15 primary factors of personality. There is evidence that the same may apply to FFM inventories [59]. Measurement invariance is thus another reason to measure sex differences at the level of narrow traits, instead of focusing on broad traits like the Big Five.

#### Univariate Versus Multivariate Effect Sizes

Since personality is a multidimensional construct, the question of how to quantify the *overall* magnitude of sex differences in personality is far from trivial. A common way of dealing with multiple effect sizes is to simply average them. For Big Five traits, the average absolute effect size across studies is 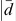 = .16 to .19, corresponding to an overlap of about 87% between the male and female distributions [11], [16]. When narrower traits are measured, average effect sizes increase somewhat. For example, Costa and colleagues [49] analyzed sex differences in FFM facets; their average effect sizes were 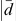 = .24 (US adults) and 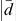 = .19 (adults from other countries). As reported above, Booth and Irwing [54] found 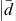 = .26 for observed scores on the 15 primary factors of the 16PF. Finally, the average effect size in Weisberg and colleagues [53] was 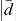 = .21 for the Big Five and 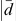 = .26 for the ten FFM aspects (uncorrected raw scores).

The problem with this approach is that it fails to provide an accurate estimate of overall sex differences; in fact, average effect sizes grossly underestimate the true extent to which the sexes differ. When two groups differ on more than one variable, many comparatively small differences may add up to a large overall effect; in addition, the pattern of correlations between variables can substantially affect the end result. As a simple illustrative example, consider two fictional towns, Lowtown and Hightown. The distance between the two towns can be measured on three (orthogonal) dimensions: longitude, latitude, and altitude. Hightown is 3,000 feet higher than Lowtown, and they are located 3 miles apart in the north-south direction and 3 miles apart in the east-west direction. What is the overall distance between Hightown and Lowtown? The average of the three measures is 2.2 miles, but it is easy to see that this is the wrong answer. The actual distance is the Euclidean distance, i.e., 4.3 miles – almost twice the “average” value.

The same reasoning applies to between-group differences in multidimensional constructs such as personality. When groups differ along many variables at once, the overall between-group difference is not accurately represented by the average of univariate effect sizes; in order to properly aggregate differences across variables while keeping correlation patterns into account, it is necessary to compute a *multivariate* effect size. The Mahalanobis distance *D* is the natural metric for such comparisons. Mahalanobis' *D* is the multivariate generalization of Cohen's *d*, and has the same substantive meaning. Specifically, *D* represents the standardized difference between two groups along the discriminant axis; for example, *D* = 1.00 means that the two group centroids are one standard deviation apart on the discriminant axis. A crucial (and convenient) property of *D* is that it can be translated to an overlap coefficient in exactly the same way as *d*: for example, two multivariate normal distributions overlap by 50% when *D* = .85, just as two univariate normal distributions overlap by 50% when *d* = .85 [60], [61]. The only difference between *d* and *D* is that the latter is an unsigned quantity. The formula for *D* is
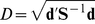
(1)where **d** is the vector of univariate standardized differences (Cohen's *d*) and **S** is the correlation matrix. Confidence intervals on *D* can be computed analytically [61], [62] or bootstrapped. For more information about *D* and its applications in sex differences research, see [2], [63], [64].

Multivariate effect sizes can make a big difference in the study of sex differences. Del Giudice [2] reanalyzed a dataset collected by Noftle and Shaver [65] in an undergraduate sample. On the Big Five, univariate effect sizes (corrected for unreliability) ranged from *d* = −.57 to +.11, and the average absolute effect size was a “small” 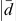 = .30, corresponding to a 79% overlap between the male and female distributions. However, the multivariate effect size was *D* = .98, a “large” effect corresponding to a multivariate overlap of 45%.

In a similar fashion, univariate effect sizes in the study by Weisberg and colleagues [53] ranged from *d* = −.49 to +.07, and the average absolute effect size on the ten FFM aspects was 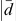 = .29 (corrected for unreliability). Computing a multivariate effect size with the same scores, however, gives *D* = .94, corresponding to an overlap of 47%. The importance of using multivariate effect sizes is further increased by the fact that personality traits interact with each other to determine behavior [66]; for example, high extraversion can have very different consequences when coupled with high versus low agreeableness. For this reason, global, “configural” sex differences (quantified by multivariate effect sizes such as *D*) may be especially relevant in determining both the social perception and the social behavior of the two sexes.

#### Summary

Based on the evidence discussed in this section, we advance the following guidelines for the accurate quantification of sex differences in personality: (a) measure personality with a higher resolution than that afforded by the Big Five (or other factors at the same hierarchical level); (b) estimate sex differences on latent factors rather than observed scores; and (c) assess global differences between males and females by computing multivariate effect sizes such as *D*. Of course, it may or may not be possible to follow all these guidelines in any given study; still, they can help researchers select the best analytic strategy in the light of the available data.

To our knowledge, none of the empirical studies published so far satisfies all these criteria. In particular, there is only one instance in which MG-CMSA has been used to estimate mean differences in an omnibus measure of personality [54], and only one instance in which global sex differences were measured with multivariate effect sizes [2].

## Methods

### Participants and Measures

The current study utilized the 1993 US standardization sample of the 16PF, 5^th^ Edition (N = 10,261), which has been previously analyzed by Booth and Irwing [54]. The sample is structured to be demographically representative of the general population of the USA. Participants were 50.1% female (N = 5,137) and 49.9% male (N = 5,124). The sample is primarily white (77.9%; N = 7,994), is proportionally geographically distributed and on average, the educational level and years in education of the sample is greater than that of the US population.

The 16PF 5^th^ Edition (16PF5) contains 185 items organized into 16 primary factor scales [67]. The 16PF5 contains 15 primary personality scales, a 15-item Reasoning scale, and a 12-item Impression Management Scale. The current analysis utilizes the 15 personality scales: Warmth (reserved vs. warm), Emotional Stability (reactive vs. emotionally stable), Dominance (deferential vs. dominant), Liveliness (serious vs. lively), Rule-Consciousness (expedient vs. rule-conscious), Social Boldness (shy vs. socially bold), Sensitivity (utilitarian vs. sensitive), Vigilance (trusting vs. vigilant), Abstractness (grounded vs. abstracted), Privateness (forthright vs. private), Apprehension (self-assured vs. apprehensive), Openness to Change (traditional vs. open to change), Self-Reliance (group-oriented vs. self-reliant), Perfectionism (tolerates disorder vs. perfectionistic), and Tension (relaxed vs. tense). The internal consistency of the 15 scales (α) ranged from .68 to .87 (see Table 1).

**Table 1:** Correlations and univariate effect sizes for observed scores.

	A.	C.	E.	F.	G.	H.	I.	L.	M.	N.	O.	Q1.	Q2.	Q3.	Q4.
A. Warmth		.16	.14	.35	.07	.44	.17	−.09	−.07	−.32	−.03	.03	−.38	−.05	−.10
C. Em. Stab.	.17		.15	.16	.25	.33	−.09	−.39	−.35	−.11	−.52	.05	−.28	.10	−.41
E. Dominance	.16	.22		.17	−.08	.37	−.10	.04	.01	−.21	−.23	.25	−.10	.09	.09
F. Liveliness	.29	.15	.15		−.08	.44	−.04	.01	.08	−.27	−.07	.11	−.47	−.12	−.05
G. Rule-Con.	.09	.36	.06	−.11		.02	−.08	−.07	−.39	.10	−.12	−.27	−.11	.30	−.26
H. Soc. Bold.	.50	.40	.34	.40	.17		−.04	−.14	−.05	−.39	−.31	.19	−.31	.00	−.20
I. Sensitivity	.17	−.24	−.14	−.04	−.22	−.06		−.07	.18	−.08	.15	.10	.11	−.06	.05
L. Vigilance	−.15	−.38	.01	.03	−.18	−.19	−.02		.19	.22	.23	−.07	.16	.06	.28
M. Abstract.	−.08	−.48	−.12	.06	−.47	−.19	.38	.27		−.07	.22	.37	.15	−.37	.20
N. Privateness	−.38	−.12	−.17	−.28	.01	−.41	−.10	.20	−.02		.01	−.11	.27	.13	.00
O. Apprehens.	−.06	−.61	−.22	−.10	−.21	−.34	.23	.28	.36	.05		−.09	.14	−.01	.36
Q1. Open. Ch.	.07	−.06	.16	.11	−.29	.07	.28	−.02	.40	−.15	.04		.01	−.15	−.10
Q2. Self-Rel.	−.38	−.36	−.17	−.46	−.17	−.42	.19	.19	.25	.29	.27	.05		.10	.23
Q3. Perfect.	−.02	.20	.18	−.10	.40	.13	−.19	−.01	−.40	.05	−.09	−.18	−.03		−.08
Q4. Tension	−.17	−.46	.01	−.08	−.34	−.33	.08	.32	.30	.13	.45	.01	.30	−.19	
*d*	−.50	+.32	+.27	−.03	+.11	+.06	−1.34	−.03	−.04	+.20	−.59	−.04	−.06	+.03	−.24
α	.69	.79	.68	.73	.77	.87	.79	.73	.78	.77	.80	.68	.79	.74	.79

*Note*. Correlations above the diagonal = females (N = 5,137); below the diagonal = males (N = 5,124). Positive effect sizes indicate that males score higher than females. Correlations and effect sizes are uncorrected (i.e., not adjusted for score unreliability).

The 15 primary scales can be further organized into 5 global scales: Extraversion (Warmth, Liveliness, Social Boldness, Privateness, and Self-Reliance), Anxiety (Emotional Stability, Vigilance, Apprehension, and Tension), Tough-Mindedness (Warmth, Sensitivity, Abstractedness, and Openness to Change), Independence (Dominance, Social Boldness, Vigilance, and Openness to Change) and Self-Control (Liveliness, Rule-Consciousness, and Perfectionism. The global scales of the 16PF are similar to the 5 FFM domains; in particular, Extraversion overlaps considerably with FFM extraversion, Anxiety with Neuroticism, Self-Control with Conscientiousness, and Tough-Mindedness with (negative) Openness. The Independence scale, however, has no clear-cut analogue in the FFM [68].

### Data analysis

Booth and Irwing [54] estimated the univariate effect sizes for both the observed scores and latent variables of the primary personality scales in the 16PF. In the current analysis, we utilize these estimates along with the correlation matrices derived from the male and female groups, for both observed scores and latent variables. A brief description of the estimation procedure is presented here (for details see [54]).

Latent mean scores were estimated using multi-group covariance and mean structure analysis (MG-CMSA). In MG-CMSA, invariance constraints are placed on a series of parameters in order to test the equivalence of the model across groups. In accordance with the suggestions of Widaman and Reise [58], invariance in the pattern of the factor loadings (configural), the degree of factor loadings (metric) and the intercepts of indicators (scalar) were tested. Booth and Irwing [54] considered changes of equal to or less than −0.01 for CFI, 0.013 for the RMSEA and −0.008 for the NNFI to support invariance. Applying these criteria, invariance was supported for the 15 primary personality scales of the 16PF. Means and variances were taken from the output of the scalar invariant model; observed score estimates were calculated based on observed facet scale scores calculated in accordance with the 16PF5 test manual [67].

Values of *D*, confidence intervals, and overlap coefficients were computed in R 2.11 [69]. We followed Reiser's exact method for confidence intervals [61], [62]. The R script was written by one of the authors (MDG) and can be downloaded at http://bsb-lab.org/site/wp-content/uploads/mahalanobis.zip. Correlation matrices for males and females were pooled prior to computing *D* (see [2]). The equivalence of the correlation matrices for males and females was established by adding an additional invariance constraint on the correlations between the latent variables in the scalar invariant model (Table 1, Model 3b in [54]). This resulted in only a minor decrease in model fit (ΔRMSEA = 0.004; ΔCFI = −0.01; ΔNNFI = −0.01) according to the criteria applied by Booth and Irwing [54]. Thus, it was concluded that the assumption of invariance in the correlations between the latent variables held across the sexes, and that the male and female matrices could be meaningfully pooled in the calculation of multivariate effect sizes.

## Results

Univariate effect sizes and correlations between personality factors are shown in Table 1 (observed scores) and Table 2 (latent variables). Univariate *d*'s were the same as reported in Booth and Irwing [54]; the average absolute effect was 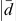 = .26 for observed scores and 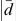 = .44 for latent variables. Correcting observed scores for unreliability raised the average absolute effect size to 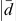 = .29. In univariate terms, the largest differences between the sexes were found in Sensitivity, Warmth, and Apprehension (higher in females), and Emotional stability, Dominance, Rule-consciousness, and Vigilance (higher in males). These effects subsume the classic sex differences in instrumentality/expressiveness or dominance/nurturance (see [11]).

**Table 2:** Correlations and univariate effect sizes for latent variables.

	A.	C.	E.	F.	G.	H.	I.	L.	M.	N.	O.	Q1.	Q2.	Q3.	Q4.
A. Warmth		.22	.18	.52	.09	.49	.32	−.15	−.07	−.52	.00	.15	−.57	−.07	−.19
C. Em. Stabil.	.25		.22	.15	.26	.41	−.14	−.48	−.42	−.18	−.76	.16	−.40	.12	−.61
E. Dominance	.18	.33		.29	−.07	.52	−.16	.09	.03	−.21	−.33	.38	−.17	.13	.08
F. Liveliness	.43	.16	.22		−.26	.57	.00	.09	.19	−.40	−.08	.17	−.60	−.23	−.02
G. Rule-Con.	.15	.44	.11	−.26		−.01	−.12	−.10	−.50	.08	−.08	−.35	−.12	.50	−.32
H. Soc. Bold.	.52	.52	.49	.50	.19		−.06	−.15	−.01	−.51	−.43	.33	−.43	.00	−.25
I. Sensitivity	.30	−.35	−.25	.00	−.29	−.09		−.14	.24	−.11	.18	.23	.13	−.01	.10
L. Vigilance	−.25	−.48	.05	.11	−.26	−.22	−.05		.24	.32	.32	−.22	.22	.07	.40
M. Abstract.	−.08	−.62	−.17	.14	−.62	−.23	.48	.35		−05	.30	.49	.20	−.51	.26
N. Privateness	−.58	−.21	−.18	−.37	−.05	−.55	−.11	.32	.05		.06	−.20	.43	.15	.05
O. Apprehens.	−.07	−.84	−.31	−.10	−.24	−47	.30	.40	.49	.13		−.18	.21	−.03	.49
Q1. Open. Ch.	.25	−.01	.22	.16	−.35	.17	.47	−.12	.51	−.23	.03		−.06	−.21	−.20
Q2. Self-Rel.	−.54	−.51	−.29	−.56	−.25	−.55	.22	.27	.36	.46	.36	.01		.10	.36
Q3. Perfect.	−.05	.25	.25	−.22	.61	.16	−.26	−.01	−.56	.04	−.12	−.25	−.08		−.09
Q4. Tension	−.31	−.68	−.02	−.08	−.42	−.40	.13	.43	.41	.25	.58	−.04	.42	−.21	
*d*	−.89	+.53	+.54	−.05	+.39	+.18	−2.29	+.36	−.01	+.15	−.60	+.21	−.12	+.04	−.27

*Note*. Correlations above the diagonal = females (N = 5,137); below the diagonal = males (N = 5,124). Positive effect sizes indicate that males score higher than females.

The uncorrected multivariate effect size for observed scores was *D* = 1.49 (with 95% CI from 1.45 to 1.53), corresponding to an overlap of 29%. Correcting for score unreliability yielded *D* = 1.72, corresponding to an overlap of 24%. The multivariate effect for latent variables was *D* = 2.71 (with 95% CI from 2.66 to 2.76); this is an extremely large effect, corresponding to an overlap of only 10% between the male and female distributions (assuming normality).

On the basis of univariate *d*'s (Table 2), it might be hypothesized that global sex differences are overwhelmingly determined by the large effect size on factor I, or Sensitivity (*d* = −2.29). Thus, we recomputed the multivariate effect size for latent variables excluding Sensitivity; the remaining *d*'s ranged from −.89 to +.54. The resulting effect was *D* = 1.71 (with 95% CI from 1.66 to 1.75), still an extremely large difference implying an overlap of 24% between the male and female distributions (the corresponding effect size for observed scores, corrected for unreliability, was *D* = 1.07, implying a 42% overlap). In other words, the large value of *D* could not be explained away by the difference in Sensitivity, as removing the latter caused the overlap between males and females to increase by only 14%. While Sensitivity certainly contributed to the overall effect size, the large magnitude of global sex differences was primarily driven by the other personality factors and the pattern of correlations among them. It should be noted that Sensitivity is not a marginal aspect of personality; in the 16PF questionnaire, Sensitivity differentiates people who are sensitive, aesthetic, sentimental, intuitive, and tender-minded from those who are utilitarian, objective, unsentimental, and tough-minded. This factor overlaps considerably with “feminine openness/closedness”, identified by Costa and colleagues [49] as a cross-culturally stable dimension of sex differences in personality.

## Discussion

Any meaningful debate on the origins of sex differences in personality needs a firm grounding in accurate empirical data [36]. It is therefore crucial to obtain good estimates of global sex differences in personality, but doing so requires researchers to address a number of methodological challenges. In this paper, we reviewed those challenges and advanced a set of guidelines for the accurate quantification of sex differences. We then applied these guidelines to the analysis of a large, representative dataset – the 1993 US validation sample of the 16PF personality questionnaire.

The results were striking: the effect size for global sex differences in personality was *D* = 2.71, an extremely large effect by any psychological standard, corresponding to a 10% overlap between the male and female distributions (assuming normality). Even removing the variable with the largest univariate effect size (Sensitivity), the multivariate effect was *D* = 1.71 (24% overlap assuming normality). These effect sizes firmly place personality in the same category of other psychological constructs showing large, robust sex differences, such as aggression and vocational interests. Global sex differences in aggression, computed on observed scores across measurement methods, range from about *D* = .89 to *D* = 1.01 [2]; vocational interests show strong sex differentiation along the “people-things” dimension, with observed effect sizes consistently around *d* = 1.2 [11].

**Figure 1:**
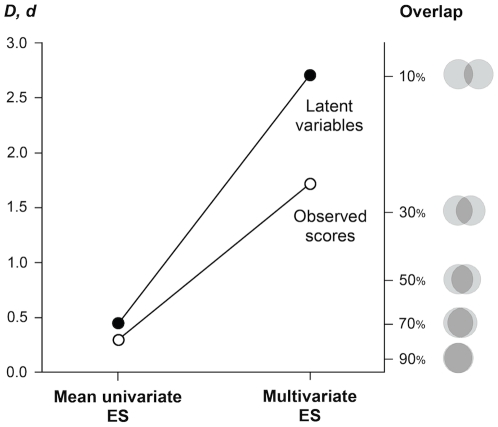
The magnitude of global sex differences in personality, estimated with different methods from the same dataset. The effect size (ES) increases dramatically as better methods are employed. The male-female overlap (right-hand axis) is calculated on the joint distribution assuming multivariate normality.

It is especially interesting to consider how effect sizes increase as better data-analytic methods are employed (Figure 1). When observed scores were used and univariate effect sizes were aggregated by simply averaging them (the weakest methodology), the overall male-female difference was “small” and consistent with Hyde's meta-analytic results [9]. However, when univariate effect sizes were estimated on latent variables and aggregated in a multvariate index (the strongest methodology), sex differences increased about tenfold and became extremely large. The idea that, on average, there are only minor differences between the personality profiles of males and females should be rejected as based on an inadequate methodology. For example, a recent analysis of FFM aspects by Weisberg and colleagues [53] led the authors to conclude that sex differences in personality are small to moderate, and that the distributions of men and women are largely overlapping. However, the analysis relied on observed scores, and the authors did not aggregate univariate differences into a proper multivariate effect size. When we computed a multivariate difference based on observed scores, it turned out to be fairly large (*D* = .94). Latent variable modeling of the same data would provide an even more accurate measure of true score differences and, given the evidence presented in the current study, would likely result in a larger overall effect size.

### Possible Objections

We anticipate three main objections to our present findings. First of all, it could be argued that the guidelines used to interpret the magnitude of univariate effect sizes (*d*) do not apply to multivariate effect sizes (*D*). However, this objection is invalid, because the substantive interpretation of *D* is exactly the same as that of *d*. Indeed, a given value of *D* or *d* indicates precisely the same statistical overlap between distributions [60], and statistical overlap is commonly used to substantiate the interpretation of effect sizes (e.g., [9]). Thus, the same set of guidelines must apply to *d* and *D*, which makes *D* an especially convenient index of multivariate differences.

Another possible objection is that our findings are based on self-reported personality, and may be inflated by gender-stereotypical or socially desirable responding. Of course, the same objection would apply to virtually all of the published literature onn sex differences in personality, including Hyde's meta-analysis [9]. We consider this objection to be weak for two main reasons. First, meta-analytic evidence shows that sex differences in aggression (a highly sex-typed behavior) are very similar when assessed by observation and self-reports, and even *stronger* when measured by peer-reports [70]. Second, self-reports will actually *deflate* sex differences if people tend to rate their own personality in relation to members of their own sex instead of “people in general” [11], [71]. Indeed, if people used the mean of their own sex as a reference point, any absolute mean difference between the sexes would simply disappear from self-reported scores. Thus, more gender-stereotypical attitudes can actually lead to smaller sex differences in self-reported personality; this paradoxical effect is a likely explanation of the fact that sex differences in self-reported personality and interests are larger in more gender-egalitarian cultures [11].

A third (partial) counter-argument may be that, even if multivariate effect sizes are extremely large, the gender similarities hypothesis still applies at the level of *univariate* sex differences. This, however, is only true for observed scores (Table 1); here, only 3 out of 15 effects are “moderate” or “large” by Hyde's conventional criteria. But when more reliable effect sizes are computed on latent variables (Table 2), 7 out of 15 effects become “moderate” or “large.” Even so, tallying univariate differences is an especially poor way of quantifying global sex differences in multivariate constructs such as personality. As discussed above, many comparatively small effects on different variables can add up to a large overall effect; moreover, the only proper way to take correlations among variables into account is to compute a multivariate index such as Mahalanobis' *D*.

### Conclusion

In conclusion, we believe we made it clear that the true extent of sex differences in human personality has been consistently underestimated. While our current estimate represents a substantial improvement on the existing literature, we urge researchers to replicate this type of analysis with other datasets and different personality measures. An especially critical task will be to compare self-reported personality with observer ratings and other, more objective evaluation methods. Of course, the methodological guidelines presented in this paper can and should be applied to domains of individual differences other than personality, including vocational interests, cognitive abilities, creativity, and so forth. Moreover, the pattern of global sex differences in these domains may help elucidate the meaning and generality of the broad dimension of individual differences known as “masculinity-femininity” [11]. In this way, it will be possible to build a solid foundation for the scientific study of psychological sex differences and their biological and cultural origins.
